# Human amniotic membrane mesenchymal stem cells-conditioned medium attenuates myocardial ischemia-reperfusion injury in rats by targeting oxidative stress

**DOI:** 10.22038/ijbms.2020.47572.10952

**Published:** 2020-11

**Authors:** Behnaz Mokhtari, Yaser Azizi, Aliakbar Rostami Abookheili, Nahid Aboutaleb, Donya Nazarinia, Nasim Naderi

**Affiliations:** 1 Physiology Research Center, Iran University of Medical Sciences, Tehran, Iran; 2 Department of Physiology, Faculty of Medicine, Iran University of Medical Sciences, Tehran, Iran; 3 Shefa Neuroscience Research Center, Khatam Alanbia Hospital, Tehran, Iran; 4 Department of Physiology, School of Paramedical Sciences, Dezful University of Medical Sciences, Dezful, Iran; 5 Rajaie Cardiovascular Medical and Research Center, Iran University of Medical Sciences, Tehran, Iran

**Keywords:** Conditioned medium, Ischemic heart diseases, Mesenchymal stem cells, Myocardial ischemia/-reperfusion injury, Oxidative stress

## Abstract

**Objective(s):**

Ischemic heart diseases (IHD) are one of the major causes of death worldwide. Studies have shown that mesenchymal stem cells can secrete and release conditioned medium (CM) which has biological activities and can repair tissue injury. This study aimed to investigate the effects of human amniotic membrane mesenchymal stem cells (hAMCs)-CM on myocardial ischemia/reperfusion (I/R) injury in rats by targeting oxidative stress.

**Materials and Methods:**

Male Wistar rats (40 rats, weighing 200–250 g) were randomly divided into four groups: Sham, myocardial infarction (MI), MI + culture media, and MI + conditioned medium. MI was induced by ligation of the left anterior descending coronary artery for 30 min. After 15 min of reperfusion, intramyocardial injections of hAMCs-CM or culture media (150 μl) were performed. At the end of the experiment, serum levels of cardiac troponin-I (cTn-I), myocardial levels of malondialdehyde (MDA), superoxide dismutase (SOD), and glutathione peroxidase (GPx), as well as cardiac histological changes were evaluated.

**Results:**

HAMCs-CM significantly decreased cTn-I and MDA levels and increased SOD and GPx activities (*P*<0.05). In addition, hAMCs-CM improved cardiac histological changes and decreased myocardial injury percentage (*P*<0.05).

**Conclusion:**

This study showed that hAMCs-CM has cardioprotective effects in the I/R injury condition. Reduction of oxidative stress by hAMCs-CM plays a significant role in this context. Based on the results of this study, it can be concluded that hAMCs-CM can be offered as a therapeutic candidate for I/R injury in the future, but more research is needed.

## Introduction

Ischemic heart diseases (IHD) are a leading cause of death worldwide. Ischemia is a condition in which myocardial blood flow is impaired by partial or complete occlusion of the coronary artery, which can lead to myocardial cell death (1, 2). Following an acute myocardial infarction (MI), reperfusion is done in order to prevent cell necrosis and apoptosis. But reperfusion of ischemic myocardium causes myocardial reperfusion injury (3, 4). Because of the prevalence of IHD and the importance of treating these diseases, understanding signaling pathways and factors involved in ischemia/reperfusion (I/R) injury and then intervening with them are very important. Among them, oxidative stress can be mentioned (5, 6). 

Studies have shown that reperfusion of the myocardial ischemic region leads to increased myocardial oxygenation and reactive oxygen species (ROS) production (7). ROS as one of the most important mediators of I/R injury initiates reactions with biomolecules and disrupts myocardial function by activating intracellular proteolytic enzymes and induction of cell death by affecting mitochondrial permeability transition pores (MPTP) (8). The imbalance between ROS production and antioxidant defense systems including superoxide dismutase (SOD), catalase, glutathione (GSH), and glutathione peroxidase (GPx) in cardiomyocytes leads to cellular degradation and oxidative stress (9). It has been shown that oxidative stress is associated with excessive ROS production and DNA, lipid and protein modifications and ultimately activates stress signaling pathways, which leads to heart failure following I/R injury (10). For that reason, prevention of oxidative stress has attracted more attention as a target for cardioprotection against I/R injury (11).

In recent years, mesenchymal stem cells (MSCs) therapy has been suggested as a beneficial therapeutic approach in various diseases, but its exact mechanisms are still under debate (12). Studies have shown that angiogenic and anti-apoptotic effects of MSCs are obtained without long term MSCs transplantation because MSCs can release paracrine factors called conditioned medium conditioned medium (CM), and can exert beneficial effects by conditioned medium (13). Conditioned medium (containing various groups of soluble proteins and peptides) has a set of biological activities and accelerates the production of progenitor cells, stimulates angiogenesis, and reduces apoptosis or inflammation. It has been proven that conditioned medium released from MSCs can protect the heart, maintain cardiac metabolism, and stimulate neovascularization through activation of cardiac progenitor cells (14). Recently, it has been reported that MSCs of fetal origin can be isolated from the amniotic membrane of the human placenta. The human placenta can be obtained in any delivery and is often considered hospital waste, so it is easily available. Also, human amniotic membrane mesenchymal stem cells (hAMCs) show a wide phenotypic and cellular plasticity and are tolerated immunologically and can differentiate into competent cardiomyocytes *in vitro* and regenerate heart tissue *in vivo* (15). But paracrine characteristics of hAMCs have not been fully investigated in depth (14). 

Despite the advances in MI treatment, IHD are still the most important causes of death worldwide (16). By considering that oxidative stress plays an important role in I/R injury pathophysiology, finding new therapeutic strategies that protect the heart against I/R injury by targeting oxidative stress signaling pathways is very necessary. Collectively, by considering the potentials of conditioned medium obtained from hAMCs in cardiovascular medicine, we hypothesized that hAMCs-CM might reduce I/R injury through affecting oxidative stress. Therefore, we investigated the effects of intramyocardial injection of hAMCs-CM on myocardial injury marker, oxidative stress factors, cardiac histological changes, and myocardial injury percent in a rat model of myocardial I/R injury.

## Materials and Methods

***Selection of animals***

Male Wistar rats (40 rats, body weight: 200–250 g) were purchased from the Animal Laboratory of Iran University of Medical Sciences. The animals were housed in an animal room at controlled temperature (22 ± 2 °C) and humidity (55 %) with 12 hr dark and 12 hr light cycle and given free access to water and standard laboratory food. They were adapted for at least 7 days before the experiments. The procedures were performed in accordance with the Guide for the Care and Use of Laboratory Animals published by National Research Council’s criteria (NIH publication No. 86-23, revised 1985) and approved by the local animal care committee. Animal Ethical Committee of Iran University of Medical Sciences approved all experiments and protocols. 

***Isolating of MSCs***

MSCs isolation was performed according to our previous study (17). Briefly, the amniotic membrane was isolated from decidua tissue. After washing the amniotic membrane several times with cold PBS, vessels and blood clots were eliminated. Then amniotic membranes were cut into small parts by a mechanical technique. After homogenizing small parts of amniotic membranes and centrifuging for 5 min at 1,250 rpm and removing supernatants, 30 ml collagenase was added to the pellet and incubated at 37 °C for 60 min in a humidified 5 % CO_2_ incubator. Then samples were centrifuged at 1,250 rpm for 5 min and supernatants were removed. Next, the pellets were exposed to trypsin 0.25 % (containing 1 ml EDTA) and incubated at 37 °C in 5 % CO_2_ for 30 min. In order to remove the red blood cells, samples were washed and then treated with Tris-ammonium chloride for 10 min. In the final step, appropriate cell density was gained via resuspension of pellet in an appropriate volume in Dulbecco’s modified eagle media (DMEM, Gibco Company, New York, USA), supplemented with 10 % fetal bovine serum (FBS, Gibco Company, New York, USA).

***MSCs identification***

For analyzing cultured MSCs, we used fluorescence-activated cell sorting (FACS). In brief, cultured MSCs were trypsinized and then incubated with 10 µg/ml fluorescein isothiocyanate (FITC)- or phycoerythrin (PE)-conjugated mouse monoclonal antibodies (Dako Company, USA) in PBS per 1×10^6 ^cells at 25 °C in the dark for 20 min. Antibodies that we used in this study were as follow: pan-leukocyte marker FITC-conjugated mouse anti-human CD45, MSC marker PE-conjugated mouse anti-human CD90, and MSC marker FITC-conjugated mouse anti-human CD44. Ultimately, after fixation of samples in 1% paraformaldehyde solution (Sigma Company, St. Louis, MO, USA), a flowcytometer (Partec Pas III, Germany) was used for quantifying analysis.

***Adipogenic and osteogenic differentiation evaluation***

For proving adipogenic differentiation capability of MSCs, MSCs were incubated for 3 weeks with an adipogenic induction medium (consisting of 50 µM indomethacin, 10^-6^ M dexamethasone, 10 µg/ml insulin, and 100 µg/ml 3-isobutyl-1-methylxanthine). Oil red O staining was performed for observing the development of some lipid droplets in cultured MSCs. 

For checking osteogenic differentiation capability of MSCs, MSCs were treated for 4 weeks with an osteogenic induction medium containing DMEM, 10 % FBS, 50 µg/ml ascorbic acid, 10 mM glycerophosphate disodium, and 10^-7^ M dexamethasone. Von Kossa staining was performed for observing calcium deposits. 

***Conditioned medium preparation***

First, MSCs were cultured at 1×10^6^ cells/cm^2^ in a medium containing α-MEM supplemented with 10 % FBS, 100 U/ml penicillin, 2 mM L-glutamine, and 100 µg/ml streptomycin. In passage 3, the medium was replaced with DMEM without antibiotics and FBS. Next, the cells were incubated in a hypoxic incubator (1% O_2_, 5% CO_2_, and 94 % N_2_) for 48 hr. In order to remove cell debris or detached cells, conditioned medium harvested from hypoxic MSCs was sucked from the dish and centrifuged at 1,200 rpm for 10 min. Then conditioned medium was filtered from a 0.22 µm filter and stored at -80 °C prior to experiments.

***Rat model of myocardial I/R***

**Figure 1:**
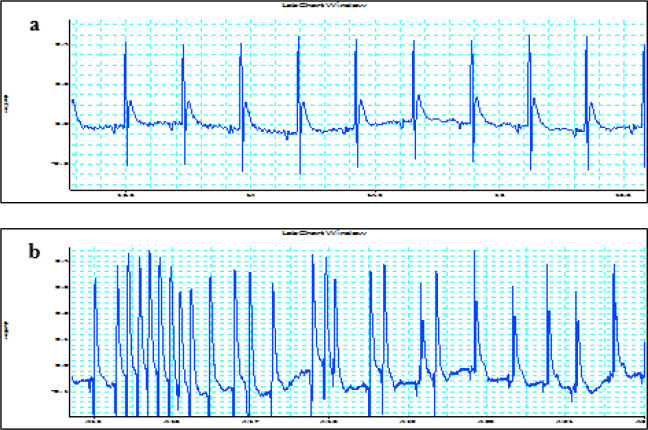
Representative ECG recordings from the Sham and MI groups. Similar results were observed in at least 38/40 rats. a) Standard ECG trace from a rat heart of the Sham group; b) Typical arrhythmias in a rat heart from the MI group. ECG: Electrocardiogram, MI: Myocardial Infarction

MI was induced by ligation of the left anterior descending coronary artery (LAD). First, the rats were anesthetized with intraperitoneal injection of a mixture of ketamine (60 mg/kg) and xylazine (5 mg/kg). Then the animals were placed in the supine position and body temperature was maintained as close as possible to 37 °C by means of a thermal pad and heating lamp. Then, they were intubated and ventilated by room air using a rodent ventilator with 2-3 ml tidal volume and 65–70/min respiratory rate (Harvard Apparatus VentElit, USA). An intercostal thoracotomy in the left fourth intercostal space was performed under sterile conditions. When the heart was exposed, the pericardium was incised. A 6.0 silk suture was passed under the LAD in approximately 1-2 mm distal from its origin and the LAD was occluded for 30 min. Standard limb lead-II electrocardiogram (ECG) was monitored continuously throughout the experimental period by an 8-channel recorder interfaced with a computer running Power Lab 8/35 data acquisition system (ADInstruments, Australia) and Lab Chart software. ECGs were recorded by using three-needle electrodes placed subcutaneously. Successful constriction of LAD was characterized by cyanosis of the affected myocardium and ECG changes consisting of ST segment elevation immediately after ligation (Figure 1). 30 min after LAD occlusion, the LAD ligature was released and reperfusion to the ischemic region was confirmed by restoration of bright red tissue color and presence of arrhythmias monitored by ECG trace. After completion of all surgical procedures, the chest was closed in layers with 2.0 silk suture and the lungs were inflated by increasing positive end-expiratory pressure. Once animals started making attempts to breathe spontaneously, the animals were removed from the ventilator and allowed to recover. The sham-operated rats underwent the same procedure of thoracotomy, without ligation of LAD. 

***Experimental design***

The animals were divided into 4 groups (n=10 per group) as follows:

1. Group I: sham-operated group (Sham) in which the animals underwent the same procedure of thoracotomy, without ligation of LAD;

2. Group II: MI group in which the animals were subjected to 30 min regional ischemia and 24 hr reperfusion; 

3. Group III: MI group plus culture media (MI + culture media) in which the animals were subjected to 30 min regional ischemia. After 15 min reperfusion, 150 µl culture media was injected into 3 different sites of the infarct border zone followed by 24 hr reperfusion; 

4. Group IV: MI group plus conditioned medium (MI + conditioned medium) in which the animals were subjected to 30 min regional ischemia. After 15 min reperfusion, 150 µl conditioned medium was injected into 3 different sites of the infarct border zone followed by 24 hr reperfusion.

**Figure 2:**
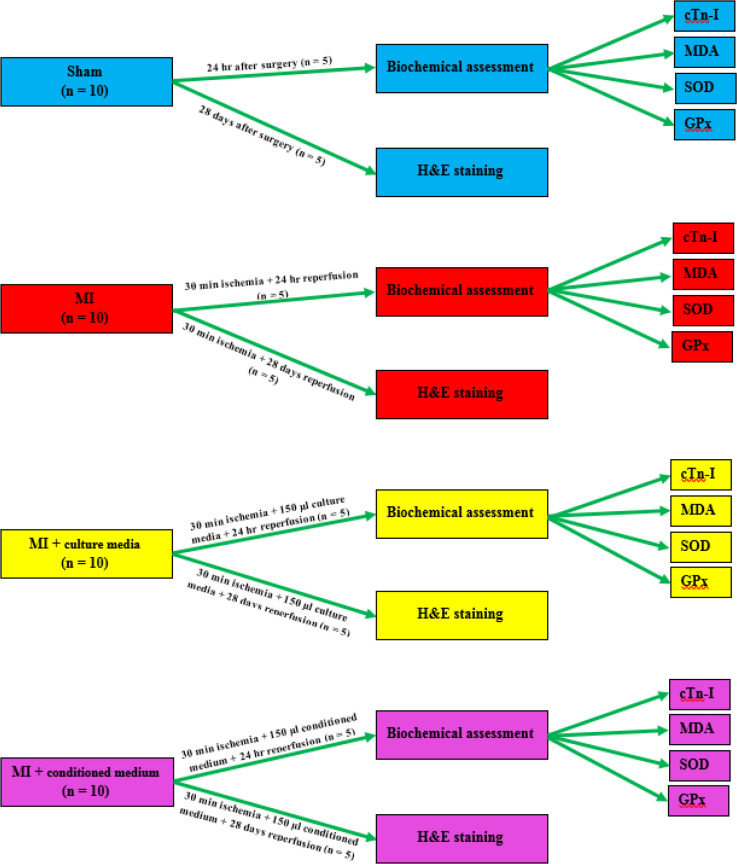
Outline scheme of experimental design

After 24 hr reperfusion, blood sampling was performed in 5 rats per group for measuring serum levels of cardiac troponin-I (cTn-I). Then left ventricles (LVs) were used for biochemical assessments. The remaining rats (n=5 per group) were followed for 28 days after reperfusion and then were sacrificed for evaluation of cardiac histological changes (Figure 2).

***Blood sampling and tissue preparation for biochemical analysis***

After 24 hr reperfusion, the rats (n = 5 per group) were anesthetized with intraperitoneal injection of ketamine (60 mg/kg) and xylazine (5 mg/kg). Sternotomies were performed and blood samples were collected from the hearts. Then the hearts were rapidly removed and after washing in normal saline, tissues from the LVs of the Sham group, and peri-infarct area and border zone of LVs in MI, MI + culture media, and MI + conditioned medium groups were rapidly frozen in liquid nitrogen and stored at -80 °C for further biochemical assessments. Blood samples were centrifuged for 15 min at 5,000 rpm, 4 °C. Then the serums were removed and stored at -80 °C until biochemical assessments.

***Measurement of cTn-I levels***

Serum levels of cTn-I as a myocardial injury marker were measured by a specific kit purchased from Monobind Inc. (Lake Forest, California, USA) according to the manufacturer’s instructions. The recorded values for cTn-I were presented in ng/ml.

***Measurement of MDA levels***

MDA levels in the myocardium were determined by a modified version of the method described by Ohkawa *et al* (18). Hearts were homogenized in 10 % trichloroacetic acid at 4 °C. A 0.2 ml homogenate was pipetted into a test tube, followed by the addition of 0.2 ml of 8.1 % sodium dodecyl sulfate (SDS), 1.5 ml of 20 % acetic acid (pH-3.5), and 1.5 ml of 0.8 % thiobarbituric acid (TBA). Tubes were boiled for 60 min at 95 °C and then cooled on ice. 1.0 ml double distilled water and 5.0 ml of n-butanol: pyridine (15:1 v/v) mixture were added to the tubes and centrifuged at 4,000×g for 10 min. Color absorbance in the organic layer was measured at 532 nm. Thiobarbituric acid reactive substances (TBARS) level was determined from the standard curve of TBA adduct formation when various concentrations of commercially available 1,1,3,3-tetraethoxypropane (Sigma chemical company, USA) were subjected to the above procedure. MDA level was expressed as nM/g wet weight.

***Measurement of SOD activity***

Myocardial SOD activity was determined using the McCord and Fridovich method (19) and modified by Kakkar *et al* (20). The hearts were homogenized in 0.25 M Tris sucrose buffer and then centrifuged for 15 min at 10,000×g, 4 °C. The supernatant was fractionated by 50 % ammonium sulfate and mixed vigorously and the reaction mixture was incubated at 4 °C for 4 hr. After centrifuging the samples at 10,000×g for 30 min at 4 °C, the supernatant was kept overnight for dialysis in 0.0025 M Tris HCl buffer. Aliquots of the supernatant (100 µl) were added to sodium pyrophosphate buffer (pH-8.3) followed by addition of 0.1 ml of 186 µl phenazine methosulphate, 0.3 ml of 300 mM nitroblue tetrazolium, and 0.2 ml of 780 µl NADH. After incubating the reaction mixture for 90 sec at 30 °C, the reaction was stopped by adding 1.0 ml of acetic acid. Then 4.0 ml of n-butanol was added and centrifuged at 3,000×g for 10 min. Organic layer absorbance was measured at 560 nm. SOD activity was determined by the standard curve obtained by using known concentrations of commercially available SOD (Sigma chemical company, USA), subjected to the above treatment. Myocardial SOD activity was presented as I.U/mg protein as compared with the standard. Protein concentration was measured by the Bradford method.

***Measurement of GPx activity***

Myocardial GPx activity was determined by the method described by Paglia and Valentine (21) and modified by Wendel (22). Hearts were homogenized at 4 °C in 0.25 M phosphate buffer saline (pH-7.0) and then centrifuged for 60 min at 15,000×g, 4 °C, and supernatant was assayed for GPx activity. GPx activity was assayed in a 1.0 ml cuvette containing 400 μl of 0.25 M potassium phosphate buffer (pH-7.0), 100 μl of 10 mM GSH, 100 μl of 2.5 mM NADPH, and 100 μl of glutathione reductase (6 U/ml). The reaction was started by adding 100 μl of 12 mM hydrogen peroxide and changes of absorbance were measured at 366 nm at 1 min interval for 5 min. GPx activity was expressed as I.U/mg protein as compared with the standard. Protein concentration was measured by the Bradford method. 

***Hematoxylin and eosin (H&E) staining***

On the 28^th^ day of reperfusion, animals (n=5 per group) were sacrificed under deep anesthesia. After performing sternotomies, the hearts were removed and washed in normal saline. Next, the hearts were fixed in 10 % neutral buffered formalin for 24–48 hr and then embedded in paraffin. Transverse sections were prepared from paraffin-embedded hearts by microtome and stained with H&E (Sigma-Aldrich Co., MO, USA) for histological examination. Then, they were observed under a light microscope with high magnification (Labomed Inc., USA) and photographs were prepared with a DeltaPix microscope camera. The severity of the cardiac injury was scored based on these variables: necrosis, interstitial edema, cardiac intercellular spaces, myofibrillar thinning, and wavy pattern consistent with infiltration and transmigration of inflammatory cells. The degree of microscopic injury of the heart was semi-quantitatively evaluated and described and was graded on a scale of 0–4 with 0= no injury; 1= injury to 25% of the field; 2= injury to 50 % of the field; 3= injury to 75% of the field; and 4= severe injury. All these assays were performed in a blinded manner. Each slide was studied at least twice. 

***Statistical analysis***

All the data were expressed as mean±standard deviation (SD). Data analysis was carried out using SPSS statistical software. One-way analysis of variance (ANOVA) was used to compare differences between the experimental groups followed by the Tukey *post hoc* test. A value of *P*<0.05 was considered to be statistically significant.

## Results

***MSC characterization***

**Figure 3:**
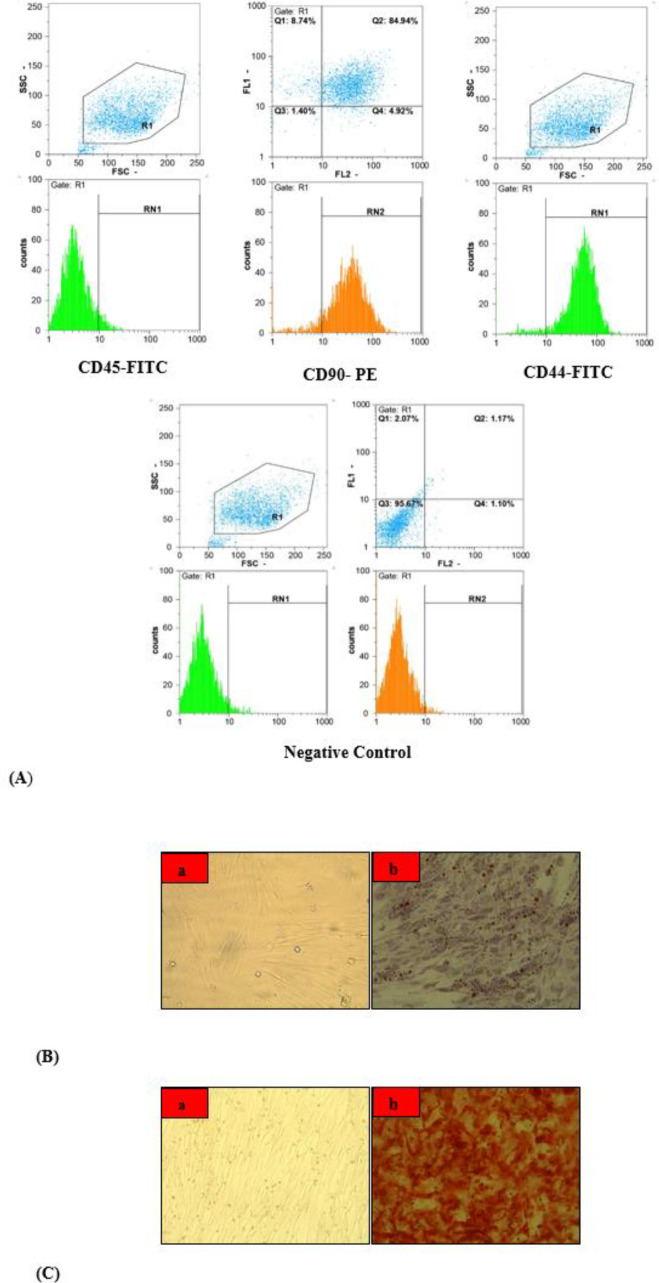
Flow cytometric analysis for expression of Mo Ab CD45-FITC, Mo Ab CD90-PE, and Mo Ab CD44-FITC (A). Oil red O staining showed development of some lipid droplets in cultured MSCs. a) Cultured MSCs; b) Adipogenic differentiation of MSCs (B). Von Kossa staining showed formation of mineralized matrix in cultured MSCs. a) Cultured MSCs; b) Osteogenic differentiation of MSCs (C). Mo Ab: Monoclonal Antibody, FITC: Fluorescein Isothiocyanate, PE: Phycoerythritin, MSCs: Mesenchymal Stem Cells

The cultured cells were expanded until passage 4 and a confluent monolayer of fibroblast-like MSCs was shaped. As shown in Figure 3A, FACS analysis showed that the pan-leukocyte marker (monoclonal antibody CD45-FITC) did not show significant expression, whereas MSCs markers (monoclonal antibodies CD90-PE and CD44-FITC) were highly expressed in cultured cells. These findings indicated a highly purified MSCs isolation. As shown in Figure 3B, Oil red O staining proved the ability of MSCs to differentiate into adipocytes by showing the development of some lipid droplets in cultured MSCs. In addition, von Kossa staining showed the ability of MSCs to differentiate into osteocytes by showing the formation of a mineralized matrix in cultured MSCs (Figure 3C).

***Effects of hAMCs-CM on cTn-I serum levels***

**Figure 4:**
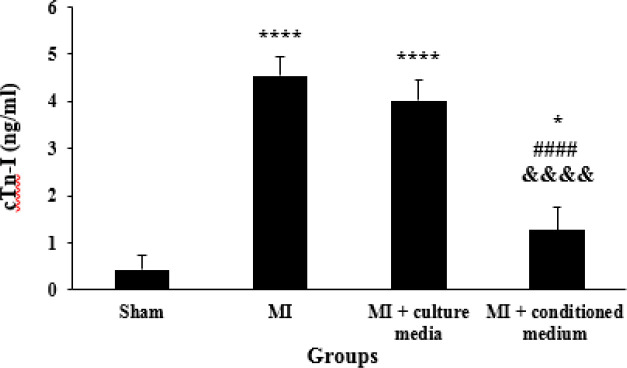
Serum levels of cTn-I in different groups (n=5 for each group). The data were expressed as Mean±SD. (*P<0.05 and **** *P*<0.0001 vs Sham group, *P*<0.0001 vs MI group, &&&& *P*<0.0001 vs MI+culture media group). cTn-I: Cardiac Troponin-I, SD: Standard Deviation, MI: Myocardial Infarction

The serum levels of cTn-I in MI and MI + culture media groups were significantly higher than those of the Sham group (*P*<0.0001). Treatment with hAMCs-CM decreased serum levels of cTn-I as compared with MI and MI + culture media groups (*P*<0.0001). On the other hand, there was a significant difference between MI + conditioned medium and Sham groups (*P*=0.023). There was no significant difference between MI and culture media treated animals in serum levels of cTn-I (*P*=0.24, Figure 4).

***Effects of hAMCs-CM on myocardial levels of MDA, SOD, and GPx***

**Figure 5:**
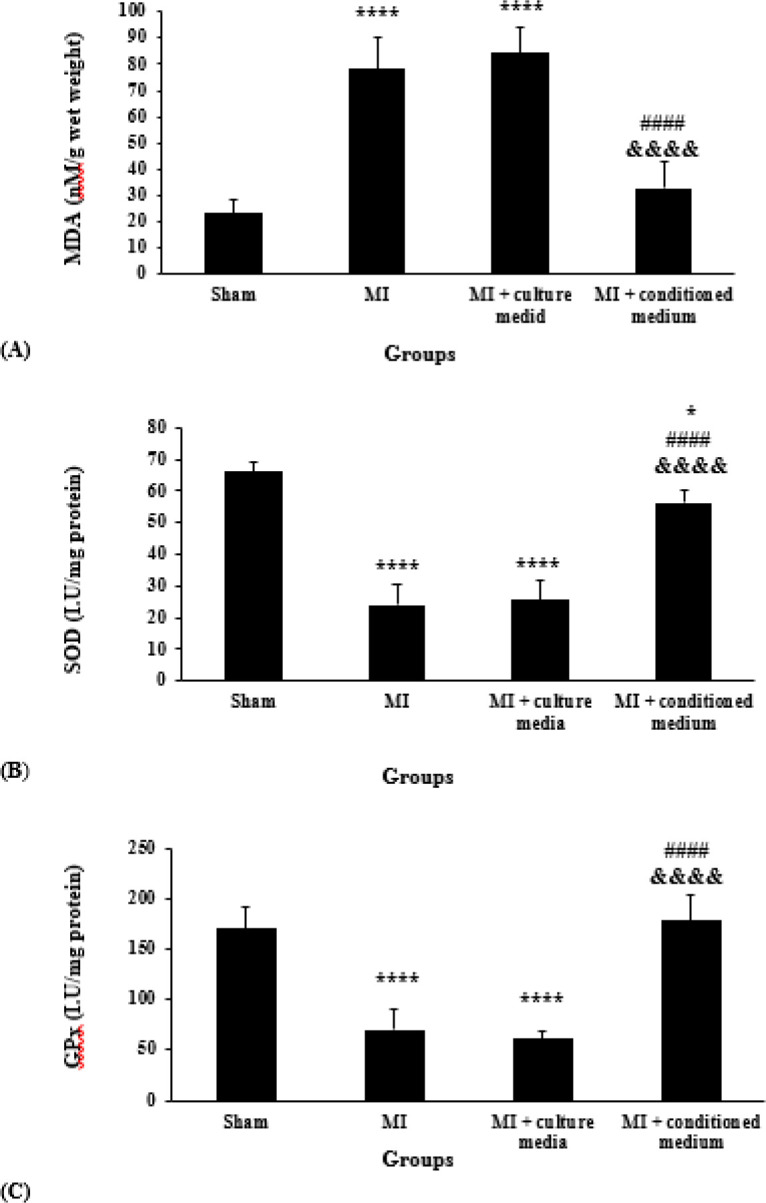
Myocardial contents of MDA (A); SOD (B); and GPx (C) in different groups (n=5 for each group). The data were expressed as Mean±SD. (* *P*<0.05 and **** *P*<0.0001 vs Sham group, *P*< 0.0001 vs MI group, &&&&*P*<0.0001 vs MI + culture media group). MDA: Malondialdehyde, SOD: Superoxide Dismutase, GPx: Glutathione Peroxidase, SD: Standard Deviation, MI: Myocardial Infarction

MDA was significantly increased in MI and MI + culture media groups as compared with the Sham group (*P*<0.0001). Treatment with hAMCs-CM significantly decreased MDA levels as compared with MI and MI + culture media groups (*P*<0.0001). Statistical analysis also showed that there was no significant difference between MI + conditioned medium and Sham groups (*P*=0.386). Furthermore, MDA was still high in the treatment group with culture media and there was no significant difference between MI and MI + culture media groups (*P*=0.738, Figure 5A).

The activity of SOD was significantly decreased in MI and MI + culture media groups as compared with the Sham group (*P*<0.0001). However, the activity of SOD in the MI + conditioned medium group was higher than in MI and MI + culture media groups (*P*<0.0001). On the other hand, there was a significant difference between MI + conditioned medium and Sham groups (*P*=0.04). Furthermore, SOD was low in MI + culture media group and there was no significant difference between MI and MI + culture media groups (*P*=0.967, Figure 5B). 

Figure 5C shows that the activity of GPx was significantly decreased in MI and MI + culture media groups compared with the Sham group (*P*<0.0001). In addition, our results showed that GPx activity in MI + conditioned medium group was higher than MI and MI + culture media groups (*P*<0.0001). There was no significant difference between MI + conditioned medium and Sham groups (*P*=0.924). Also, GPx activity was still low in the MI + culture media group and there was no significant difference between MI and MI + culture media groups (*P*=0.882, Figure 5C).

***Effects of hAMCs-CM on cardiac histological changes***

**Figure 6:**
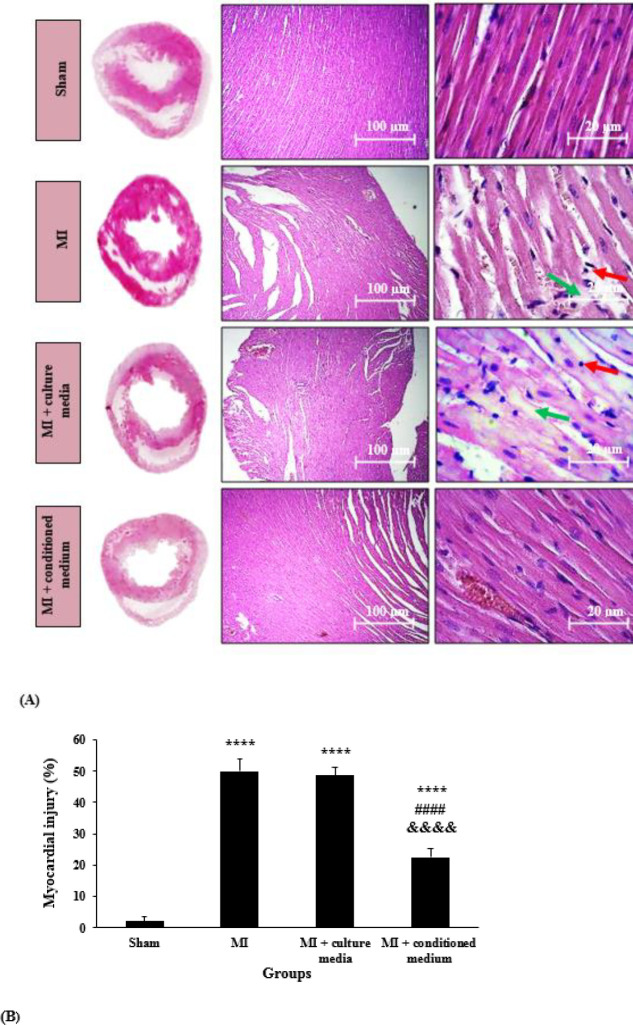
Cross-sections of the middle part of left ventricle tissue stained with hematoxylin and eosin in different groups (original magnification from left to right 4× and 40×, respectively). Red arrows indicate lymphocyte cells and green arrows indicate necrosis (A). Semi-quantitative analysis of myocardial injury score (n=5 per group). The data were expressed as mean±SD. ( ****P<0.0001 vs Sham group, *P*<0.0001 vs MI group, &&&&*P*<0.0001 vs MI + culture media group) (B). SD: Standard Deviation, MI: Myocardial Infarction

H&E staining was used for the evaluation of cardiac histological changes in different groups. As shown in Figure 6A, in the Sham group, cardiomyocytes were arranged in rows, the fiber structure of cardiomyocytes was clear, there was no damage to cardiac fibers, and the cytoplasm was uniform. Moreover, there was no evidence of necrosis or the infiltration of lymphocyte and other inflammatory cells. However, acute ischemic changes including necrosis, interstitial edema, increasing in cardiac intercellular spaces, myofibrillar thinning, and wavy pattern consistent with infiltration and transmigration of inflammatory cells, which indicate reperfusion injury, all were observed in the MI group. These changes were lower in MI + conditioned medium group. However, treatment with culture media could not improve the mentioned morphological changes.

Semi-quantitative analysis of myocardial injury score (Figure 6B) showed that myocardial injury score in MI and MI + culture media groups was significantly higher as compared with the Sham group (*P*<0.0001). HAMCs-CM could significantly reduce myocardial injury score compared with MI and MI + culture media groups (*P*<0.0001). Furthermore, there was a significant difference between MI + conditioned medium and Sham groups (*P*<0.0001). However, treatment with culture media could not significantly reduce myocardial injury score as compared with the MI group (*P*=0.927).

## Discussion

In the present study, we investigated the effects of conditioned medium derived from hAMCs on cardiac I/R injury in coping with oxidative stress in a rat model of MI. Parameters that were evaluated in our study contain serum levels of cTn-I and myocardial oxidative stress factors (MDA, SOD, and GPx), 24 hr after reperfusion. To better evaluate the effects of hAMCs-CM on myocardial I/R injury, we assessed cardiac histological changes on the 28^th^ day of reperfusion. The results of our study showed that treatment with hAMCs-CM significantly reduced the increased levels of MDA and increased the activities of SOD and GPx following MI. On the other hand, decreased serum levels of cTn-I and improvement of morphological changes in the heart tissue were also evident for protective effects of hAMCs-CM in cardiac I/R injury.

Previous experimental studies have reported the protective effects of different MSCs-derived conditioned medium in MI models. Since oxidative stress has a key role in the pathophysiology of cardiac I/R injury, investigating new therapeutic strategies having positive effects against oxidative damage is very important. Thus modulation of oxidative stress by hAMCs-CM might be a novel approach to protect against myocardial I/R injury. For that reason, we investigated the effects of hAMCs-CM on oxidative stress in a rat model of myocardial I/R. By evaluating endpoints, we found that I/R caused enhancement of cTn-I levels and cardiac tissue injury. It is proven that the most specific and sensitive marker of myocardial injury is cTn-I, which is released into the blood from the cytosol of myocytes in cardiac injury conditions such as I/R. So cTn-I is a specific marker of acute MI and myocardial I/R injury (23). By evaluating this endpoint, we found that I/R caused enhancement of cTn-I levels. In addition, our morphological analysis showed that I/R led to interstitial edema, necrosis and infiltration, and transmigration of inflammatory cells. Interestingly, injection of hAMCs-CM decreased the levels of cTn-I, as well as, reduced cardiac tissue injury, which was proven by improving all of the above-mentioned criteria. Our study proved that these positive effects of the conditioned medium in I/R injury were associated with its beneficial effects on oxidative stress through reduction of MDA as a marker of lipid peroxidation, and elevation in SOD and GPx activities as antioxidant markers. 

Oxidative stress is a major cause of myocardial damage following I/R injury. Studies have shown that in I/R condition, dysfunctional mitochondria lead to oxidative stress and consequently heart failure and other cardiovascular diseases. Besides, defective mitochondrial electron transport chain causes ROS generation which activates cytotoxic mechanisms responsible for apoptosis and necrosis. In addition, ROS may cause mitochondrial damage and cytochrome c releasing, caspases activation, and apoptosis. It has been demonstrated that following MI, oxidative stress is increased in both infarcted and non-infarcted myocardium (7, 24). In accordance with previous studies, we also established that 30 min ischemia and 24 hr reperfusion led to a significant increase in myocardial MDA levels and a significant decrease in SOD and GPx activities. So it can be concluded that I/R injury caused an imbalance between ROS production and antioxidant defense systems in cardiomyocytes, which led to cellular damage and oxidative stress. Interestingly, intramyocardial injection of hAMCs-CM significantly decreased MDA levels and increased SOD and GPx activities. So it can be said that hAMCs-CM exerts its cardioprotective effect against I/R injury by making a balance between ROS production and inhibition.

In agreement with our study, Timmers *et al. *demonstrated that human embryonic stem cells-conditioned medium (hESCs-CM) can stimulate angiogenesis and increase vascular density, thereby reduce infarct size and improve systolic and diastolic function in a porcine model of MI. They also demonstrated that hESC-CM exerts its protective effects by reducing active caspase-3, probably through decreasing oxidative stress and modulation of TGF-β signaling (13, 25). Another study showed that administration of dental pulp derived conditioned medium protected the heart from ischemia through two mechanisms. These mechanisms include reduction of cardiomyocyte apoptosis and inhibition of inflammatory response by reducing the expression of IL-6, IL-1β, and TNF-α in cardiomyocytes (26). Furthermore, *in vitro* and *ex vivo* experiments showed that MSCs-CM infusion at the onset of cardiac reperfusion following ischemia can reduce I/R injury and infarct size by activating the PI3K pathway (27). On the other hand, a study established that hAMCs-CM protects cardiac-like cells from hypoxia/reoxygenation injury by inhibition of apoptosis. Moreover, *in vivo* experiment showed that hAMCs-CM activates ERK 1/2 MAPK pathway and inhibits proapoptotic pathways such as SAPK/JNK and p38 MAPK (14).

The results of the previous studies mentioned above, are very similar to our findings in the MI model in rats. Our study established that hAMCs produce and release factors that are capable to repair myocardial damage after I/R injury. likewise, we found that hAMCs-CM has protective effects in myocardial I/R injury condition and reduces myocardial injury by preventing oxidative stress. According to the results of our study, intramyocardial injection of hAMCs-CM after 15 min of reperfusion caused significant protective effects in I/R injury condition by reduction of MDA levels and elevation of SOD and GPx activities. 

According to previous studies and our findings, it seems that hAMCs-CM may decrease oxidative stress through inhibition of ROS, inflammation, and pro-apoptotic signaling pathways as its cardioprotective effects, but still needs more investigation. The involvement of oxidative stress was evaluated in this study. Investigating the role of signaling pathways can help us to get a precise understanding of hAMCs-CM effects in myocardial I/R injury. By considering the potentials of hAMCs-CM in cardiovascular medicine, it can be said that hAMCs-CM has beneficial effects in reducing the adverse effects of myocardial I/R injury due to its effect on oxidative stress.

As a final point, it should be noted that many drugs such as antiplatelets and anticoagulants have promising effects in MI. However, even by using the most effective drugs in MI, myocardial infarct size in many patients is still high (14). In the present work, we have demonstrated that conditioned medium derived from an ethically acceptable source of MSCs can repair infarcted hearts without any manipulation. So hAMCs-CM can be proposed as an excellent candidate for reduction of severity of myocardial I/R injury in the future, but a lot of researches are needed. It should be mentioned that due to financial limitations, we were unable to evaluate the mechanisms in which hAMCs-CM inhibits oxidative stress. However, the results of this study open up potential avenues for future studies to examine the protective effects of hAMCs-CM and its related signaling pathways in the context of cardioprotection in MI conditions. 

## Conclusion

In general, it can be concluded that one of the main mechanisms in which hAMCs-CM exerts its protective effects against I/R injury is inhibition of oxidative stress. Based on the results of this study, hAMCs-CM can be proposed as a candidate for reduction of severity of myocardial I/R injury in the future, but a lot of researches are needed. 

## References

[1] Hosseini L, Vafaee MS, Badalzadeh R (2020). Melatonin and nicotinamide mononucleotide attenuate myocardial ischemia/reperfusion injury via modulation of mitochondrial function and hemodynamic parameters in aged rats. J Cardiovasc Pharmacol Ther.

[2] Badalzadeh R, Yousefi B, Tajaddini A, Ahmadian N (2015). Diosgenin-induced protection against myocardial ischaemia-reperfusion injury is mediated by mitochondrial KATP channels in a rat model. Perfusion.

[3] Bayrami G, Karimi P, Agha-Hosseini F, Feyzizadeh S, Badalzadeh R (2018). Effect of ischemic postconditioning on myocardial function and infarct size following reperfusion injury in diabetic rats pretreated with vildagliptin. J Cardiovasc Pharmacol Ther.

[4] Najafi M, Noroozi E, Javadi A, Badalzadeh R (2018). Anti-arrhythmogenic and anti-inflammatory effects of troxerutin in ischemia/reperfusion injury of diabetic myocardium. Biomed Pharmacother.

[5] Al-Salam S, Hashmi S (2018). Myocardial ischemia reperfusion injury: apoptotic, inflammatory and oxidative stress role of galectin-3. Cell Physiol Biochem.

[6] Badalzadeh R, Azimi A, Alihemmati A, Yousefi B (2017). Chronic type-I diabetes could not impede the anti-inflammatory and anti-apoptotic effects of combined postconditioning with ischemia and cyclosporine A in myocardial reperfusion injury. J Physiol Biochem.

[7] González-Montero J, Brito R, Gajardo AI, Rodrigo R (2018). Myocardial reperfusion injury and oxidative stress: therapeutic opportunities. World J Cardiol.

[8] Raedschelders K, Ansley DM, Chen DD (2012). The cellular and molecular origin of reactive oxygen species generation during myocardial ischemia and reperfusion. Pharmacol Ther.

[9] Weiss S (1986). Oxygen, ischemia and inflammation. Acta Physiol Scand Suppl.

[10] Sengupta A, Molkentin JD, Paik J-H, DePinho RA, Yutzey KE (2011). FoxO transcription factors promote cardiomyocyte survival upon induction of oxidative stress. J Biol Chem.

[11] Kurian GA, Rajagopal R, Vedantham S, Rajesh M (2016). The role of oxidative stress in myocardial ischemia and reperfusion injury and remodeling: revisited. Oxid Med Cell Longev.

[12] Lai RC, Arslan F, Tan SS, Tan B, Choo A, Lee MM (2010). Derivation and characterization of human fetal MSCs: an alternative cell source for large-scale production of cardioprotective microparticles. J Mole Cell Cardiol.

[13] Timmers L, Lim SK, Arslan F, Armstrong JS, Hoefer IE, Doevendans PA (2008). Reduction of myocardial infarct size by human mesenchymal stem cell conditioned medium. Stem Cell Res.

[14] Danieli P, Malpasso G, Ciuffreda MC, Cervio E, Calvillo L, Copes F (2015). Conditioned medium from human amniotic mesenchymal stromal cells limits infarct size and enhances angiogenesis. Stem Cells Trans Med.

[15] Kimura M, Toyoda M, Gojo S, Itakura Y, Kami D, Miyoshi S (2012). Allogeneic amniotic membrane-derived mesenchymal stromal cell transplantation in a porcine model of chronic myocardial ischemia. J Stem Cells Regen Med.

[16] Huang C, Gu H, Yu Q, Manukyan MC, Poynter JA, Wang M (2011). Sca-1+ cardiac stem cells mediate acute cardioprotection via paracrine factor SDF-1 following myocardial ischemia/reperfusion. PLOS One.

[17] Tousi SMTR, Amirizadeh N, Nasirinezhad F, Nikougoftar M, Ganjibakhsh M, Aboutaleb N (2017). A rapid and cost-effective protocol for isolating mesenchymal stem cells from the human amniotic membrane. Galen Med J.

[18] Ohkawa H, Ohishi N, Yagi K (1979). Assay for lipid peroxides in animal tissues by thiobarbituric acid reaction. Anal Biochem.

[19] McCord JM, Fridovich I (1969). Superoxide dismutase an enzymic function for erythrocuprein (hemocuprein). J Biol Chem.

[20] Kakkar P, Das B, Viswanathan P (1984). A modified spectrophotometric assay of superoxide dismutase. Indian J Biochem Biophys.

[21] Paglia DE, Valentine WN (1967). Studies on the quantitative and qualitative characterization of erythrocyte glutathione peroxidase. J Lab Clin Med.

[22] Wendel A (1981). Glutathione peroxidase. Methods Enzymol.

[23] Al-Mallah M, Zuberi O, Arida M, Kim HE (2008). Positive troponin in diabetic ketoacidosis without evident acute coronary syndrome predicts adverse cardiac events. Clin Cardiol.

[24] Sun Y (2007). Oxidative stress and cardiac repair/remodeling following infarction. Am J Med Sci.

[25] Timmers L, Lim SK, Hoefer IE, Arslan F, Lai RC, van Oorschot AAM (2011). Human mesenchymal stem cell-conditioned medium improves cardiac function following myocardial infarction. Stem Cell Res.

[26] Yamaguchi S, Shibata R, Yamamoto N, Nishikawa M, Hibi H, Tanigawa T (2015). Dental pulp-derived stem cell conditioned medium reduces cardiac injury following ischemia-reperfusion. Sci Rep.

[27] Angoulvant D, Ivanes F, Ferrera R, Matthews PG, Nataf S, Ovize M (2011). Mesenchymal stem cell conditioned media attenuates in vitro and ex vivo myocardial reperfusion injury. J Heart Lung Transpl.

